# Three different ways of training ultrasound student-tutors yield significant gains in tutee’s scanning-skills

**DOI:** 10.3205/zma001285

**Published:** 2019-11-15

**Authors:** Nora Celebi, Jan Griewatz, Madeleine Ilg, Stephan Zipfel, Reimer Riessen, Tatjana Hoffmann, Nisar Peter Malek, Jan Pauluschke-Fröhlich, Ines Debove, Reinhold Muller, Eckhart Fröhlich

**Affiliations:** 1 PHV dialysis center, Waiblingen, Germany; 2 Competence Centre for University Teaching in Medicine, Baden-Württemberg, Tübingen, Germany; 3 Eberhard-Karls University, Medical faculty, Tübingen, Germany; 4 University Hospital Tübingen, Department of Internal Medicine VI, Tübingen, Germany; 5 University Hospital Tübingen, Department of Internal Medicine VIII, Tübingen, Germany; 6 University Hospital Tübingen, Department of Internal Medicine I, Tübingen, Germany; 7 University Hospital Tübingen, Department of Gynecology, Tübingen, Germany; 8 University Hospital Bern, Department of Neurology, Bern, Switzerland; 9 James Cook University, Smithfield, Australia

**Keywords:** medical Student, ultrasound, sonography, student tutor

## Abstract

**Aim: **Many medical universities rely these days on trained student tutors to enable faculty-wide undergraduate ultrasound training. However, there is neither consensus on an optimal method nor any developed and agreed standard in the training of these student tutors. Usually internships and courses are employed which have both a specific set of advantages and disadvantages.

We conducted a prospective quasi-randomized study of assess the effects of three types of tutor training on the resulting improvement in scanning skills of their tutees.

**Methods: **Three batches of student tutors were trained by a course only (C-group), by an internship only (I-group) or by a course and an internship (CI-group). The respective gains in ultrasound scanning skills of the tutees were measured prospectively. A total 75 of the 124 5^th^ year medical students (60.5%) who attended the mandatory ultrasound course completed both pre- and post-exams on a voluntary basis. Within a limit of eight minutes and three images, they were asked to depict and label a maximum of 14 anatomical structures. Two blinded raters independently awarded two points for each label with an identifiable structure and one point for each label with a possibly identifiable structure.

**Results:** In all three groups, the tutees improved significantly by more than doubling their pre-score results and comparably (Gains: C-group 9.19±5.73 points, p<.0001, I-group 9.77±4.81 points, p<.0001, CI-group 8.97±5.49 points, p<.0001).

**Conclusion: **Student tutors, who were trained with a course or an internship or a course and an internship could teach scanning skills to 5th year medical students very effectively and with similar success.

## Zusammenfassung

**Ziel: **Viele medizinische Universitäten bieten heutzutage mit der Hilfe von ausgebildeten studentischen Tutoren eine fakultätsweite Ultraschall-Grundausbildung an. Es gibt jedoch weder einen Konsens über eine optimale Methode noch einen verbindlichen Standard für die Ausbildung dieser studentischen Tutoren. In der Regel werden zur Ausbildung Praktika und Kurse eingesetzt, die sowohl spezifische Vor- als auch Nachteile aufweisen.

Wir führten eine prospektive, quasi-randomisierte Studie durch, um die Auswirkungen von drei Arten von Tutorentraining auf die daraus resultierende Verbesserung der Scanfähigkeiten ihrer Tutees zu bewerten.

**Methoden:** Drei Gruppen studentischer Tutoren wurden nur durch einen Kurs (K-Gruppe), nur durch eine Famulatur (F-Gruppe) oder durch einen Kurs und eine Famulatur (KF-Gruppe) geschult. Die jeweiligen Zuwächse an Scanfähigkeiten der Tutees wurden prospektiv gemessen. 75 der 124 Medizinstudenten im fünften Studienjahr (60,5%), die den curricularen Ultraschallkurs besuchten, haben sowohl den Vor- als auch den Nachtest auf freiwilliger Basis absolviert. Innerhalb von acht Minuten sollten maximal 14 anatomische Strukturen in drei Bildern dargestellt und beschriftet werden. Zwei verblindete Bewerter vergaben unabhängig voneinander zwei Punkte für jede sicher identifizierbare Struktur und einen Punkt für jede möglicherweise identifizierbare Struktur.

**Ergebnisse:** In allen drei Gruppen verbesserten sich die Tutees signifikant, indem sie ihre Ergebnisse von vor dem Unterricht mehr als verdoppelten (Zuwachs: K-Gruppe 9,19±5,73 Punkte, p<0,0001; F-Gruppe 9,77±4,81 Punkte, p<0,0001; KF-Gruppe 8,97±5,49 Punkte, p<0,0001).

**Fazit: **Studentische Tutoren, die mit einem Kurs oder einer Famulatur oder einem Kurs und einer Famulatur geschult wurden, konnten Medizinstudenten im 5. Jahr sehr effektiv und mit vergleichbarem Erfolg Scanfähigkeiten vermitteln.

## 1. Background

Ultrasound skills are useful in clinical practice and can greatly enhance a physician’s ability to care for patients [1], [2], [3], [4], [5]. Since some skills are expected even from novice physicians [6], [7], there is a broad consent to incorporate ultrasound training into the undergraduate education [8], [9], [10]. The Task Force National Competence-Based Learning Objectives for Undergraduate Medical Education (NKLM) in Germany even mandates the teaching of ultrasound skills [http://www.nklm.de].

In order to perform ultrasound examinations, the medical students have to master theoretical knowledge and acquire the technical ability to scan and correctly interpret the ultrasound images [9], [11]. For the practical teaching most faculties rely on a hands-on training in very small groups supervised by student-tutors [12]. This concept is well accepted and it could be established that, as far as basic ultrasound skills are concerned, student-tutors are able to achieve similar teaching results as faculty members [12], [13], [14], [15].

However, data on different concepts to train student-tutors are scarce and there is no accepted standard [12]. Essentially there are two possible ways to train student-tutors: a course or an internship in an ultrasound laboratory. A course has the advantage that the instructors have full control over the content and can thus make sure that all topics the student-tutors are expected to teach in the curriculum are covered. In addition, a large number of student-tutors can be trained simultaneously. On the other hand, the time in which the students can practice the actual scanning technique is limited in a course and the organizational effort to train student-tutors is high. An internship approach to train student-tutors usually provides students with a lot of opportunity to practice and to encounter many pathologies. In this case, the organizational effort to implement a student-tutor training program is relatively low. However, there is a factor of chance and it cannot be guaranteed that all topics relevant to the curriculum are sufficiently covered in daily practice. Moreover, the number of student-tutors that can be trained in internships is limited by the capacity of the respective ultrasound laboratories. 

A combined training comprising both a course and an internship does offer training with full control over the content as well as the opportunity to learn the scanning skills hands-on and see real life pathologies. A combined training however also comes with both disadvantages described above: A substantial effort to implement the student-tutor training program and the limited capacity by the ultrasound laboratories. 

In previous studies all concepts were employed [12] but so far it remains unclear whether all concepts yield the necessary skill-gain for the tutees to teach the skill effectively. 

We trained three batches of student-tutors with either a course only (C-group), an internship only (I-group) or a course combined with an internship (CI-group). In an initial investigation we assessed the success of this training: All three teaching methods for the future student tutors resulted in significant (all p<0.001) and comparable improvements in the future tutors’ theoretical knowledge as well as in their practical scanning skills; for details please refer to [16].

However, the ultimate outcome for the student-tutor training is the skill acquisition of their tutees. Therefore, the aim of the present study was to assess prospectively, by employing a pre-post-design, whether the tutees were able to effectively improve their scanning-skills (OSCE scores) – and if so – whether this improvement was dependent on the training of their student tutors. 

## 2. Methods

### 2.1 Study design and assessment

A prospective quasi-randomized pre-post-design was employed in this trial. During the course organization the student-tutors applied for the planned lecture hours. At the commencement of course, the tutees allocated themselves among the tutors in roughly equally sized groups with a resulting tutee/tutor ratio of around 4:1. 

#### 2.2 Student-tutors

The student-tutors teaching the course were trained over four consecutive terms as follows: For two terms we concomitantly trained students by either a course only or an internship only, and for two terms the students received a combined training of a structured course and an internship. All students for the tutor teaching program were recruited from 3-5th year and accepted in the order of their application without further selection criteria. 

All student tutors sat a standardized didactical training for 1,5 days.

The course only-group (C-group) participated in a five day training program held by faculty members. The learning goals were presented with lectures followed by hands-on practicals with two students per ultrasound device and sessions of job shadowing in ultrasound laboratories. The content of the structured course can be summarized as follows: The first day covers the basic physics and the handling of the ultrasound device as well as image optimisation and the ultrasound anatomy and pathologies of liver, gallbladder, and bile ducts. On the second day, the student-tutors learned ultrasound anatomy and pathologies of the retroperitoneum, abdominal vessels, lymph nodes, pancreas, spleen, kidneys, bladder, uterus, and prostate. On the third day all the learned skills were integrated into a systematic examination of the abdomen and a special session on focused echocardiography in emergency life support (FEEL) was taught. The fourth day covered the thyroid, jugular veins, carotid arteries, and lymph nodes - again including their anatomy, scanning technique, and pathologies and duplex sonography and compression sonography of the deep veins. On the fifth and last day thoracic ultrasound and ultrasound in trauma assessment (eFAST) was taught. Overall, the students had seven hours of lecture, 21 hours of hands-on-training and 2.5 hours of job shadowing in ultrasound laboratories. The students were handed a script covering all learning goals with picture examples of pathologies.

In the laissez fair internship only-group (I-group) the students were handed the same script and asked to use it to prepare for the internship. In addition, they had a two hour meeting with a faculty member who explained the handling of the ultrasound device, orientation, artefacts and basic ultrasound anatomy. The students were free to roam through six different ultrasound laboratories for a minimum of 21 and a maximum of 35 days. The student-tutors were asked to switch rooms as soon as they felt they had mastered the learning goals of the respective ultrasound laboratory. 

In the course plus internship-group (CI-group) the future tutors sat the same course as the C-group, followed by a structured 21-day rotation through seven ultrasound laboratories (the six laboratories the students in the I-group had access to and the intensive care unit) with three weekdays for each station. The students were also handed the same script.

#### 2.3 Tutee training

The tutees were recruited from the mandatory internship internal medicine in 5th year. Although the participation in the ultrasound course was compulsive, the students were asked to participate on a voluntary basis in the anonymized objective structured clinical examination (OSCE).

The tutee training course was identical for all tutees. Prior to the course the tutees sat a lecture held by a faculty member on physics of ultrasound, artefacts, handling of the ultrasound device and orientation. The tutees were handed the same script as the student-tutors. The subsequent ultrasound course lasted 12.5 hours in total, divided into three sessions. The first and last 30-45 minutes of each course was dedicated to the OSCE.

#### 2.4 Assessment

At the beginning and the end of the course the tutees were asked to depict and label 14 anatomical structures within a timeframe of 8 minutes, using three images at most. All images were rated by two experienced sonographers who were blinded to the tutor education and the study phase (pre or post). For every label with a clearly identifiable corresponding structure on the image, the students were awarded two points. For every label with a possibly identifiable structure, the students were awarded one point. If a label was missing or the corresponding structure could not be identified, no points were given. The achieved tutees’ scores where then calculated as the average score of the two independent ratings.

The course and the assessments were conducted with four identical ultrasound devices (ACUSON X 300 PE, Version 7.0, Siemens Healthcare, Erlangen, Germany).

#### 2.5 Statistics

Sample size calculations were based on a previous publication with the same assessment [13] and revealed that 10 tutees (per group) were necessary to achieve a power in the excess of 80% to find an improvement of 7 points in their OSCE scores as significant at an alpha level of 5%. 

Inter-rater agreement was assessed by linear regression and an intraclass correlation coefficient (ICC) [17]. Categorical variables were displayed as percentages. Numerical variables proved to be reasonably normally distributed and thus means (± standard deviations (SD)) were used for descriptive purposes and supplemented by 95% confidence intervals (95% CIs) for the main outcome measures. Paired t-tests were employed for pre/post statistical comparisons within groups; one-way ANOVA was used for tests between groups. For all tests, a p-value of less than 0.05 was regarded as statistically significant. 

#### 2.6 Ethics

The project was approved by the local ethics committee of the University of Tuebingen, number 667/2016BO2. All participants gave written consent and could withdraw study participation at any time without giving any reasons. 

## 3. Results

Overall, a total of 124 students enrolled into the mandatory internship Internal Medicine program. Of those, 75 (60.5%) completed the voluntary pre- and post-OSCE and thus constitute the study sample of the tutees. The characteristics of the tutees in the three different training groups are detailed in table 1 (Tab. 1). A total of 18 tutors were trained for the program; 5 taught in the C-group; 3 in the I-Group and 10 in the CI group.

**Figure 1:**
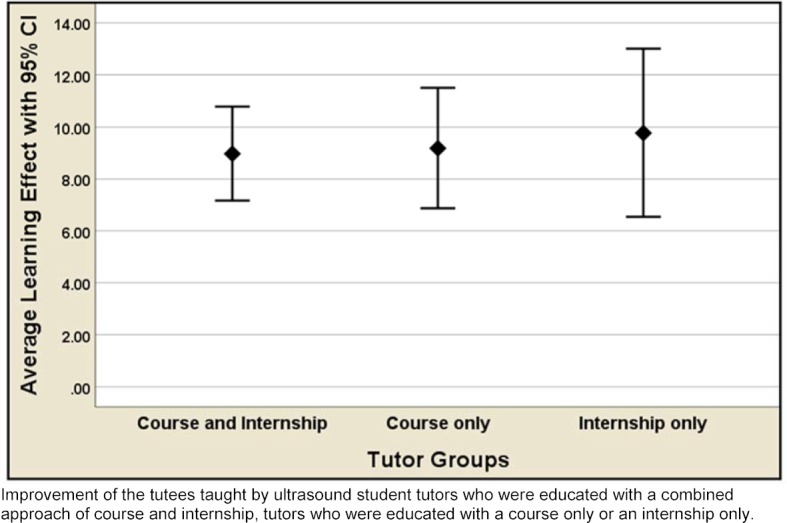
Mean Tutees’ Improvements in Scanning Skills OSCE Scores (with 95% CI) by Tutor Training Groups (p<0.001 for each group)

The inter-observer agreement between the two independent raters of the OSCE proved to be nearly ideal with a resulting linear correlation coefficient of 0.96 (p<0.001) and an ICC of 0.96 (95% CI=0.94-0.97; p<0.001) thus delivering a solid base for the OSCE scoring (see figure 1 (Fig. 1)). 

The groups were essentially comparable, only the C-group revealed slightly better pre-OSCE results than the two other groups. There was a slight, nonsignificant trend towards an older age and more ultrasound exposure in the C-group compared to the other groups. Theoretical knowledge and practical ultrasound skills, pre- and post-training, as well as the observed gains (in absolute and in relative terms) within the three groups are displayed in table 1 (Tab. 1). 

All teaching groups improved substantially, significantly, and comparably in both theoretical knowledge and practical skills (all p<0.001, paired t-tests). 

The main outcome measures (mean gains) are displayed together with their 95% confidence limits in figure 1 (Fig. 1) and table 1 (Tab. 1).

## 4. Discussion

In this study, we assessed the acquisition of scanning skills by fifth year medical students taught by student-tutors who were trained with three different teaching concepts: a laissez fair internship; a highly structured ultrasound course; or an ultrasound course followed by a highly structured 21 day rotation through seven different ultrasound-laboratories. The average gain in scanning skills was substantial and significant within each group and very similar between the groups.

This is the first study to assess the actual learning effects of the tutees taught by tutors who sat different student-tutor training concepts; previous studies nearly exclusively focused on the satisfaction with the tutors only. 

Tarique found in his extensive review that student-tutors who were trained only one week were rated inferior to faculty members, while student-tutors who were trained 2-4 weeks were rated equally [12]. Ahn and colleagues compared student-tutors from 4^th^ year and found that students,who were trained with a four-week internship were rated higher than students with a two-week internship [18]. However, the satisfaction with the student-tutors is only an indirect measure of the learning outcome. In our study, the student-tutors who were trained with a course only – and thus for one week only – achieved a significant skill improvements in their tutees comparable to the student-tutors of the I- or CI-group, who were trained for four to six weeks.

Several investigations showed that the ultrasound student-tutor concept can provide substantial and lasting scanning abilities in tutees [13], [14], [15]. Dinh reported that medical students scored nearly as high as residents in an ultrasound-scanning OSCE with a remarkable improvement compared to ultrasound-naive students, but they did not state how their tutors were trained [19]. These studies did not compare different concepts to train student-tutors. 

Another study compared the learning results of the student-tutors: Fox et al. measured the knowledge gain of students in emergency sonography and found a four-week internship superior to a two-week internship [20]. In our opinion, not only the length of the training, but also the complexity of the skills to be taught has to be taken into consideration. For more complex echocardiography skills even after a three-week internship the student-tutors did not achieve the same teaching success as faculty members [14]. On the other hand, a 30 minute training and a one week self-study phase was sufficient to train student-tutors to teach musculoskeletal ultrasound [15].

It should thus be emphasized here that our ultrasound curriculum comprises only very simple ultrasound skills. When developing the course, all involved parties agreed that only those skills that can be mastered in a very short timespan are suitable for a faculty wide ultrasound curriculum. This is because the vast majority of students do not have the opportunity for extensive supervised practice in ultrasound laboratories. 

We assessed the learning outcome under real-life conditions and on a voluntary basis; an approach which implies some limitations: first of all, only 60.5% of the students enrolled in the class completed both voluntary assessments. The students were self-allocating to their tutors, so we added some demographics to the pre-test data collection. It was found that females were under-represented in the CI-group. However gender did generally not significantly influence the learning outcome and nor did multivariate adjustment reveal any relevant changes to the displayed bivariate findings (data not shown). 

Although we aimed for a tutor/tutee-ratio of 1:4, due to tutees and tutors not showing up for class, this ratio differed slightly between the groups. 

The student-tutors in the C- and the I-group had an average of two terms more teaching experience. On the other hand, this obviously also coincided with an equally longer time period to forget the content of the initial training.

We assessed only the practical scanning skills of the tutees since the theory and the pathologies were taught with an identical lecture and script for all students and only the actual scanning was supposed to be conveyed by the student-tutors. 

The employed assessment of the scanning skills can only deliver a rather conservative measure of the actual skills: There is always some data loss on a still-frame in comparison to a moving picture; some information may be obscured by the labels; and the students also had a time- and image limit to allow for a standardization of the assessment-conditions. Thus, while the assessment was designed to provide a reliable measure for relative comparisons of the scanning skills, it is likely to underestimate the true, absolute, abilities of the students.

The study was only powered to detect relevant learning gains (>7 OSCE points) as significant within the tutees’ groups defined by different student-tutor education strategies. It was not powered to detect potential differences between these groups. For this former aim, we already had to recruit student-tutors over four consecutive terms. Sample size calculations for the latter aspect revealed that group sizes in the excess of 160 tutees per group would be necessary to detect potential subtle differences in performance between the groups (data not shown). In order to achieve this type of recruitment numbers however, the program would have to be implemented in about ten middle sized medical faculties adding not only a much longer time frame but also additional complexity from potential center-effects to the study. For these reasons, we found it prudent to settle for the more pragmatic proof of concept approach presented here and to study, whether all three approaches are worthy of further investigation.

In conclusion, under real life-conditions, 5^th^ year medical students in a mandatory faculty-wide ultrasound course were able to successfully acquire scanning skills regardless whether their student-tutors were educated by a laissez fair internship, a highly structured ultrasound course or an ultrasound course followed by highly structured 21 day rotation through seven different ultrasound-laboratories. So medical faculties striving to implement a faculty-wide ultrasound course can probably train their student-tutors in a way that optimally suits their specific conditions. Further studies are necessary to investigate, whether one training concept is superior over the others.

## 5. Declarations

### 5.1 Availability of data and material

All data generated or analysed during this study are included in this published article, missing datasets used or analysed are available from the corresponding author on reasonable request. 

#### 5.2 Competing interests 

We, the authors, declare that we have no competing interests that would influence this study. 

#### 5.3 Authors' contributions 

NC and EF conceived of the study. RM analysed the data and prepared the figures. NC, EF, JG and RM drafted and jointly prepared the manuscript. ID, JPS and MI contributed in the development of the ultrasound training concepts. RR, SZ and NM contributed to the development and implementation of the ultrasound curriculum. All authors read and approved the final manuscript. 

## Funding

We received “PROFIL”-funding from University of Tuebingen, project F.7281048 for student tutoring, statistics and translation.

## Competing interests

The authors declare that they have no competing interests. 

## Figures and Tables

**Table 1:**
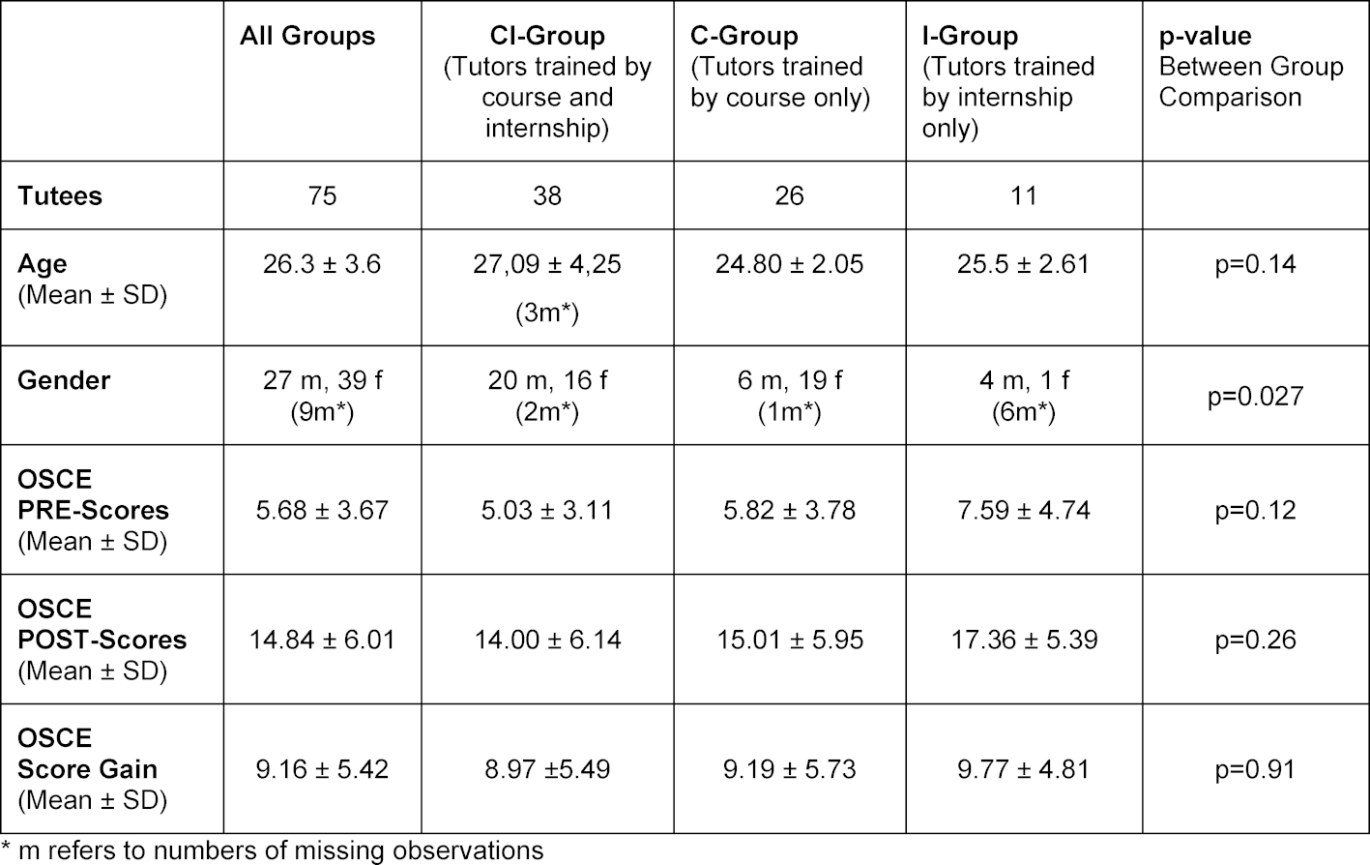
Characteristics and Scanning Skills OSCE Scores of the Tutees by Tutor Training Groups.
